# Derivation and Validation of a Scoring System to Identify Patients with Bacteremia and Hematological Malignancies at Higher Risk for Mortality

**DOI:** 10.1371/journal.pone.0051612

**Published:** 2012-12-14

**Authors:** Mario Tumbarello, Enrico Maria Trecarichi, Morena Caira, Anna Candoni, Domenico Pastore, Chiara Cattaneo, Rosa Fanci, Annamaria Nosari, Antonio Spadea, Alessandro Busca, Nicola Vianelli, Teresa Spanu, Livio Pagano, He.M.A.B.I.S. (Hematological Malignancies Associated Bacterial Infections Surveillance) Italy

**Affiliations:** 1 Istituto di Clinica delle Malattie Infettive, Università Cattolica del Sacro Cuore, Roma, Italy; 2 Istituto di Ematologia, Università Cattolica del Sacro Cuore, Roma, Italy; 3 Clinica di Ematologia, Università di Udine, Udine, Italy; 4 Divisione di Ematologia, Università di Bari, Bari, Italy; 5 U. O. Ematologia, Spedali Civili, Brescia, Italy; 6 Unità Operativa di Ematologia, Azienda Ospedaliera Universitaria Careggi, Firenze, Italy; 7 Divisione di Ematologia e Centro Trapianti Midollo, Ospedale Niguarda Ca’ Granda, Milano, Italy; 8 Ematologia, Istituto Regina Elena, Roma, Italy; 9 Divisione di Ematologia, Ospedale le Molinette, Torino, Italy; 10 Istituto di Ematologia ed Oncologia Clinica “Lorenzo e Ariosto Serágnoli”, Ospedale; S.Orsola-Malpighi, Università di Bologna, Bologna, Italy; 11 Istituto di Microbiologia, Università Cattolica del Sacro Cuore, Roma, Italy; University of Porto, Portugal

## Abstract

**Background:**

The aim of this study was to develop and validate a reliable clinical prediction rule that could be employed to identify patients at higher likelihood of mortality among those with hematological malignancies (HMs) and bacterial bloodstream infections (BBSIs).

**Methods and Findings:**

We conducted a retrospective cohort study in nine Italian hematological units. The derivation cohort consisted of adult patients with BBSI and HMs admitted to the Catholic University Hospital (Rome) between January 2002 and December 2008. Survivors and nonsurvivors were compared to identify predictors of 30-day mortality. The validation cohort consisted of patients hospitalized with BBSI and HMs who were admitted in 8 other Italian hematological units between January 2009 and December 2010. The inclusion and exclusion criteria were identical for both cohorts, with type and stage of HMs used as matching criteria. In the derivation set (247 episodes), the multivariate analysis yielded the following significant mortality-related risk factors acute renal failure (Odds Ratio [OR] 6.44, Confidential Interval [CI], 2.36–17.57, P<0.001); severe neutropenia (absolute neutrophil count <100/mm^3^) (OR 4.38, CI, 2.04–9.43, P<0.001); nosocomial infection (OR, 3.73, CI, 1.36–10.22, P = 0.01); age ≥65 years (OR, 3.42, CI, 1.49–7.80, P = 0.003); and Charlson Comorbidity Index ≥4 (OR, 3.01, CI 1.36–6.65, P = 0.006). The variables unable to be evaluated at that time (for example, prolonged neutropenia) were not included in the final logistic model. The equal-weight risk score model, which assigned 1 point to each risk factor, yielded good-excellent discrimination in both cohorts, with areas under the receiver operating curve of 0.83 versus 0.93 (derivation versus validation) and good calibration (Hosmer-Lemshow P = 0.16 versus 0.75).

**Conclusions:**

The risk index accurately identifies patients with HMs and BBSIs at high risk for mortality; a better initial predictive approach may yield better therapeutic decisions for these patients, with an eventual reduction in mortality.

## Introduction

Intensified treatment protocols with chemotherapy and/or hematological stem cell transplantation (HSCT) result in greater chances of curing patients with hematological malignancies (HMs). However, these potentially life-saving treatments increase the risk of infectious complications. Bloodstream infections (BSIs) are among the most common and severe complications observed in patients with HMs, particularly if they are neutropenic, with a prevalence ranging from 11 to 38% [1]–[6]. In addition, the onset of BSIs within 5 days of stem cell infusion has been reported in approximately 35% of patients who underwent HSCT [7].

Coagulase-negative staphylococci (CoNS), *Staphylococcus aureus*, *Enterobacteriaceae,* and *Pseudomonas aeruginosa* have been reported, at different frequencies, as the most prevalent organisms causing BSI in patients with HMs [5], [8]–[11].

The crude mortality rates for patients with BSI vary from 12% to 42%, and attributable mortality rates as high up to 30% have been reported [1], [3]–[5], [7], [9], [11]–[13]. In addition, BSIs may lead to delayed administration of chemotherapy, prolonged hospitalization, and increased costs [5], [14].

Several studies have evaluated the epidemiological and clinical characteristics of bacterial BSI (BBSIs) in patients with HMs [5], [8], [9], [11]. However, some important uncertainties remain, and to the best of our knowledge, no scoring system has yet been developed that predicts the risk of mortality in patients with HMs and concurrent BBSI.

The aim of the present study, conducted in 9 large Italian hospitals, was to develop and validate a reliable, easy-to-use, clinical prediction rule that could be employed to identify patients with higher likelihood of mortality among those with HMs and BBSI.

## Materials and Methods

### Ethics Statement

The institutional review board (Comitato Etico, Università Cattolica del Sacro Cuore) approved the study, and informed consent was waived because of the retrospecive observational nature of the study.

### Setting and Study Design

To identify risk factors for mortality in patients aged ≥18 years with HMs and BBSI, we conducted a cohort study in nine Italian hematological units. The derivation cohort consisted of patients with BBSI and HMs admitted to the Catholic University Hospital, located in Rome, between January 2002 and December 2008. Recurrent episodes of BBSI for the same patient were excluded from the study. The primary outcome measured was all-cause mortality 30 days after BBSI onset. The survivor and nonsurvivor subgroups were compared to identify predictors of 30-day mortality.

The validation cohort consisted of individuals hospitalized with BBSI and HMs who were admitted to 8 other Italian hematological units between January 2009 and December 2010. The inclusion and exclusion criteria were identical to those used for the derivation cohort, and patients included in the validation cohort were matched with those in the derivation set cohort according to type and stage of HMs.

### Definitions and Variables Analyzed

Data collected from hospital charts and the laboratory database included patient demographics, disease and disease stage at time of BBSI, type of HSCT (autologous or allogenic), medical history, clinical/laboratory findings, treatment, and outcome of infection.

The following terms were defined before the data analysis.

A BBSI was defined as an infection manifested by (I) the presence in at least 1 blood culture of bacteria other than skin contaminants (i.e., diphtheroids, *Bacillus* spp., *Propionibacterium* spp., CoNS, micrococci) or (II) the presence of any bacterial species in at least 2 consecutive blood cultures in a patient with a systemic inflammatory response syndrome [15].

The date of the 1st positive blood culture (index culture) was regarded as the date of BBSI onset.

Infections were classified as polymicrobial if 2 or more different genera were recovered from specimens drawn during the first 48 h of infection, regardless of whether the isolates came from the same or different blood culture sets.

The BBSI was classified as nosocomial if the index blood culture had been drawn more than 48 h after admission to our hospital [16]. When the index culture had been drawn within the first 48 h of hospitalization, the infection was classified as healthcare-associated or community-acquired as defined by Friedman et al. [17].

Neutropenia was defined as an absolute neutrophil count (ANC) of <500 cells/mm^3^. Neutropenia was considered prolonged if the duration was ≥10 days and severe if the ANC was <100 cells/mm^3^.

Acute renal failure was defined as a serum creatinine value >2 mg/dL in patients with previous normal renal function or an increase of >50% of the baseline creatinine level in patients with preexisting renal dysfunction.

The impact of comorbidities was determined by the Charlson Comorbidity Index [18].

### Statistical Analysis

Continuous variables were compared by Student’s *t* test for normally distributed variables and by the Mann-Whitney U test for non-normally distributed variables. Categorical variables were evaluated with the χ2 or two-tailed Fisher's exact test. Odds ratios (ORs) and 95% confidence intervals (CIs) were calculated to evaluate the strength of any association that emerged. Values are expressed as the means ± standard deviations (SD) (continuous variables) or as percentages of the group from which they were derived (categorical variables). Two-tailed tests were used to determine statistical significance; a *P* value of <0.05 was considered significant.

Variables associated with mortality in the univariate analysis (*P* ≤0.10) were included in a logistic regression model, and a backward stepwise approach was used to identify independent predictors of mortality; in addition, to develop a scoring system that could be applicable at the onset of bacteremia, the variables unable to be evaluated at that time (for example, prolonged neutropenia) were not included in the final logistic model. Variables were retained in the final model if the P value was ≤ 0.05. The final regression model was transformed into a point-based rule. An equal-weight risk score model, which assigned 1 point to each risk factor, was assessed; in addition, an unequal-weight risk score model, with weighted scores assigned to each variable obtained by dividing each regression coefficient by half of the smallest coefficient and rounding to the nearest integer, was also performed [19].

The discriminatory power of the prediction rule in the derivation group was expressed as the area under the receiver-operating characteristic curve (AUROC). An AUROC of 0.5 indicates no discriminative ability, and perfect discrimination (i.e., a test with 100% sensitivity and 100% specificity) is reflected by an AUROC of 1. An AUROC exceeding 0.8 is usually indicative of good to excellent prediction; those in the 0.7–0.8 and 0.6–0.7 ranges reflect moderate and low predictive power, respectively. The sensitivity and specificity of the prediction rule - each with 95% CIs - were calculated at different cut-off values. Positive and negative predictive values (PPV and NPV, respectively) were obtained with standard methods.

All statistical analyses were performed using the Intercooled Stata program, version 11, for Windows (Stata Corporation, College Station, Texas, USA).

## Results

**Figure 1:**
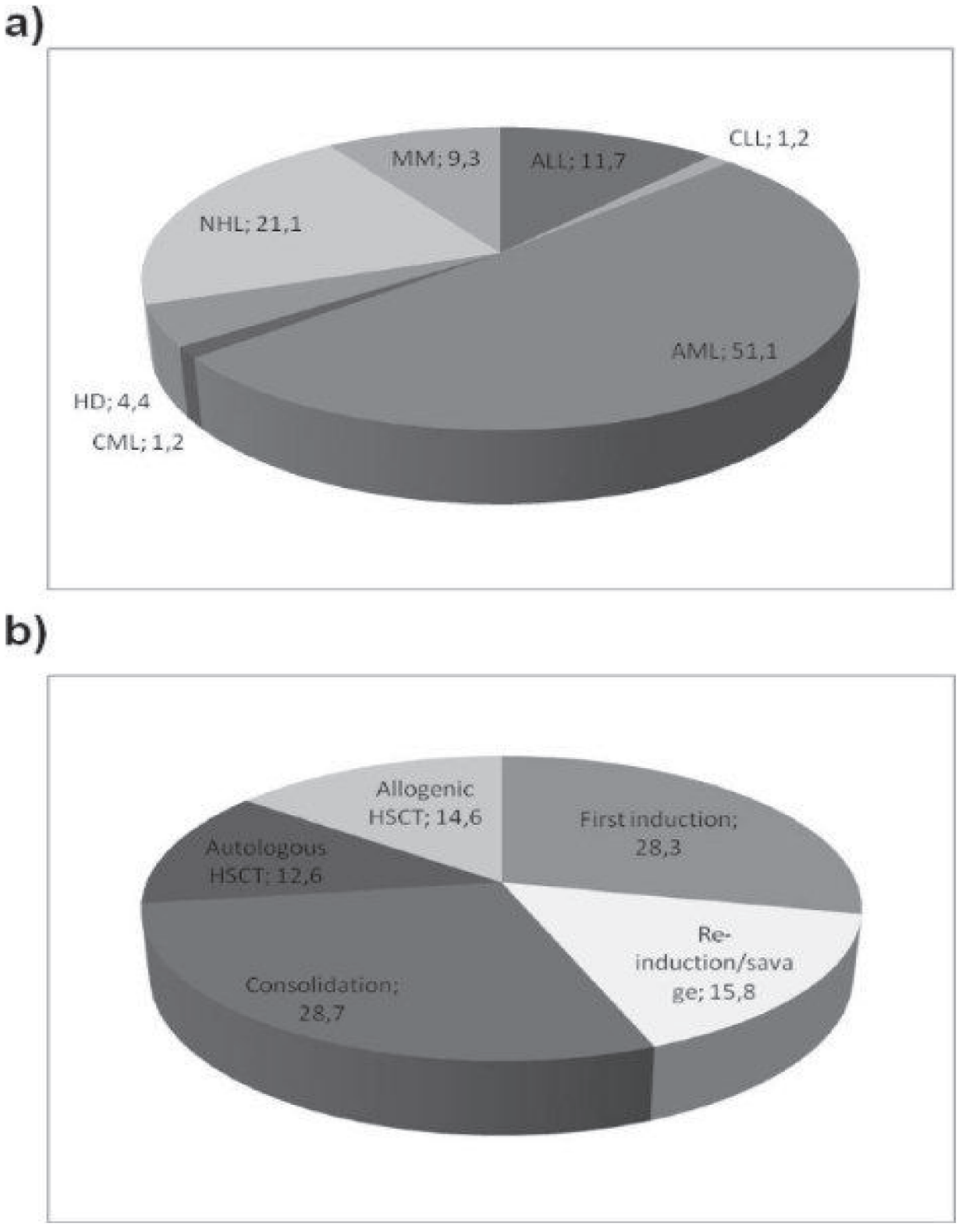
Distribution (%) of a) type of hematological malignancies and b) stages of disease in the derivation and validation sets. AML, acute myeloid leukemia; ALL, acute lymphoid leukemia; NHL, lymphoma; HD, Hodgkin’s disease; CML, chronic myeloid leukemia; MM, multiple myeloma; CLL, chronic lymphocytic leukemia; BMT, bone marrow transplantation.

Four hundred and ninety-four patients with HMs and BSI were included in the study. Figure 1 indicates the distributions of patients (both from the derivation and validation set) according to the type and stage of HMs (matching criteria).

### Derivation Cohort

Two hundred and fifty-one patients with HMs and BBSI met the inclusion criteria for the derivation cohort. Four were excluded because of missing data; thus, a total of 247 cases were included in the analysis.

Table 1 summarizes the main clinical and demographic characteristics of patients included in the derivation cohort.

**Table 1:** Comparison of characteristics of case patients in the derivation and validation groups.

Variables	No. (%) of patients		
	Derivation Set	Validation Set	
	(n = 247)	(n = 247)	
Demographic information			
Male sex	126 (51.0)	140 (56.7)	0.21
Age >65 years	60 (24.3)	47 (19.0)	0.16
Risk factors			
Charlson Comorbidity Index >4	49 (19.8)	44 (17.8)	0.56
Chronic viral hepatitis	31 (12.6)	6 (2.4)	<0.001
Chronic renal failure	6 (2.4)	7 (2.8)	0.78
Diabetes mellitus	7 (2.8)	31 (12.6)	<0.001
Receipt of corticosteroids^a^	105 (42.5)	81 (32.8)	0.02
Neutropenia	163 (65.9)	234 (94.7)	<0.001
Severe neutropenia (PMN <100/mm^3^)	87 (35.2)	144 (58.3)	<0.001
Prolonged neutropenia (≥10 days )	99 (40.1)	166 (67.2)	<0.001
Presence of central venous catheter	114 (46.2)	215 (87.0)	<0.001
Presence of urinary catheter	49 (19.8)	29 (11.7)	0.01
Presence of nasogastric tube	6 (2.4)	2 (0.8)	0.15
Total parenteral nutrition^b^	4 (1.6)	73 (29.6)	<0.001
Etiological agents			
Monomicrobial Gram-positive bacteremia	123 (55.7)	81 (38.4)	<0.001
Coagulase-negative *Staphylococcus* spp.	62 (28.1)	44 (20.8)	0.08
*Staphylococcus aureus*	22 (9.9)	7 (3.3)	0.005
*Enterococcus* spp.	15 (6.8)	7 (3.3)	0.10
*Streptococcus* spp.	11 (4.9)	12 (5.7)	0.74
Monomicrobial Gram-negative bacteremia	98 (44.3)	130 (61.6)	<0.001
*Escherichia coli*	49 (22.2)	73 (34.6)	0.004
*Klebsiella pneumoniae*	8 (3.6)	11 (5.2)	0.42
*Pseudomonas aeruginosa*	32 (14.5)	24 (11.4)	0.34
*Enterobacter* spp.	2 (0.9)	8 (3.8)	0.05
Polymicrobial bacteremia	26 (10.5)	36 (14.6)	0.17
Nosocomial bacteremia	178 (72.1)	190 (76.9)	0.22
Acute renal failure	25 (10.1)	20 (8.1)	0.43
30-day mortality	52 (21.1)	30 (12.1)	0.007

a During the 3 months preceding index blood culture.

b During the 30 days preceding index blood culture.

The most common bacterial isolates were CoNS (28.1%), *E. coli* (22.2%), *P. aeruginosa* (14.5%), and *S. aureus* (9.9%). The overall 30-day mortality rate was 21.1% (52/247) (Table 1).

The univariate analysis revealed significant differences between the survivor and nonsurvivor subgroups. A significantly higher percentage of the nonsurvivor group were ≥65 years of age (*P* = 0.007) and had nosocomial bacteremia (*P* = 0.003), indwelling urinary catheter (*P*<0.001), chronic viral hepatitis (*P* = 0.03), neutropenia (*P* = 0.02), prolonged neutropenia (*P*<0.001), severe neutropenia (*P*<0.001), Charlson Comorbidity Index ≥4 (*P*<0.001), and a clinical presentation with acute renal failure (*P*<0.001). Nonsurvivors were also more frequently treated with corticosteroids (*P*<0.001); no significant differences between survivors and nonsurvivors were observed in terms of the type of etiological agents causing BBSI, although polymicrobial BBSI was more frequent in nonsurvivors (*P* = 0.02).

In the logistic regression analysis, the five variables found to be independently associated with 30-day mortality were the following: acute renal failure (Odds Ratio [OR] 6.44, 95% confidence interval [CI], 2.36–17.57); severe neutropenia (OR 4.38, 95% CI, 2.04–9.43); nosocomial infection (OR 3.73, 95% CI, 1.36–10.22); age ≥65 years (OR 3.42, 95% CI, 1.49–7.80); and Charlson Comorbidity Index ≥4 (OR 3.01, 95% CI, 1.36–6.65) (Table 2).

**Table 2:** Multivariate logistic regression analysis of risk factors for mortality in patients with bacteremia and hematological malignancies.

Variables	P value	OR (95% CI)
Acute renal failure	<0.001	6.44 (2.36–17.57)
Severe neutropenia	<0.001	4.38 (2.04–9.43)
Nosocomial infection	0.01	3.73 (1.36–10.22)
Age ≥65 years	0.003	3.42 (1.49–7.80)
Charlson Comorbidity Index ≥4	0.006	3.01 (1.36–6.65)

### Validation Cohort

Three hundred and forty-eight patients with HMs and BBSI were observed in the 8 hospitals involved in the validation study, and 247 were selected by matching criteria with patients from the derivation set; these were included in the validation cohort. Their baseline characteristics are summarized in Table 1. Compared to the validation cohort, the derivation cohort contained a higher percentage of patients with chronic viral hepatitis (P<0.001), who had received corticosteroids (P = 0.02), and had indwelling urinary catheter (P<0.001); on the contrary, compared to the derivation cohort patients, patients included in the validation set had higher rates of diabetes mellitus (P<0.001), neutropenia (P<0.001), indwelling central venous catheter (CVC) (P<0.001), and total parenteral nutrition (P<0.001). In terms of the etiological agents causing BBSI, monomicrobial cases were caused more frequently by Gram-positive bacteria in the derivation set (P<0.001) and by Gram-negative bacteria in the validation set (P<0.001).

### Construction and Validation of the Predictive Scoring System

#### Derivation set

A scoring system that could be used to predict mortality was developed based on the independent risk factors that were identified in the multivariate analysis. An equal-weight risk score model was assessed first, assigning 1 point to each risk factor. The distributions of scores according to outcome and of variables for different score points are reported in Tables 3 and 4, respectively.

**Table 3:** Distribution of scores in the derivation and validation sets.

No. (%) of patients						
Points	Derivation Set			Validation Set		
	Nonsurvivors	Survivors	Total	Nonsurvivors	Survivors	Total
0	0	30 (100)	30	0	13 (100)	13
1	5 (5.1)	93 (94.9)	98	0	83 (100)	83
2	17 (24.3)	53 (75.7)	70	5 (4.4)	108 (95.6)	113
3	22 (61.1)	14 (38.9)	36	9 (47.4)	10 (52.6)	19
4	7 (58.3)	5 (41.7)	12	13 (81.3)	3 (18.7)	16
5	1 (100)	0	1	3 (100)	0	3
Total	52 (21.1)	195 (78.9)	247	30 (12.2)	217 (87.8)	247

**Table 4:** Distribution of variables according to score points in the derivation and validation sets.

		No. of patients					Total
Points	Population set	Variables					
		Acute renal failure	Severeneutropenia	Nosocomialinfection	Age ≥65 years	Charlson Comorbidity Index ≥4	
1	Derivation	0	10	72	13	3	98
	Validation	0	7	59	14	3	83
2	Derivation	3	42	60	22	13	70
	Validation	1	102	94	16	13	113
3	Derivation	13	24	33	16	22	36
	Validation	3	17	18	3	16	19
4	Derivation	8	10	12	8	10	12
	Validation	13	15	16	11	9	16
5	Derivation	1	1	1	1	1	1
	Validation	3	3	3	3	3	3

**Figure 2:**
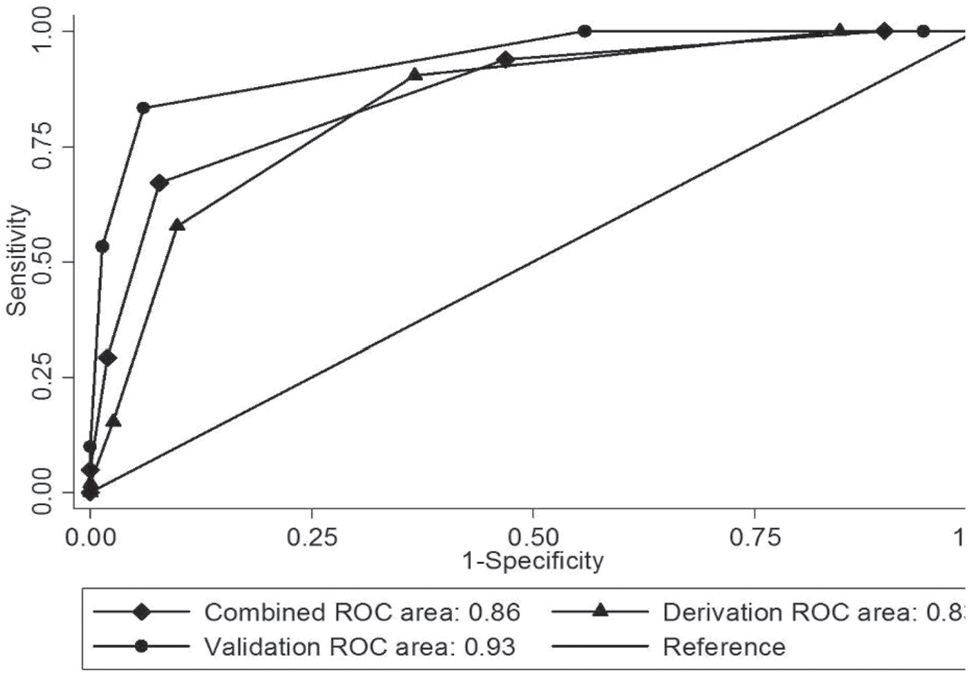
Receiver operator characteristic curves (ROC) for the scoring system in the derivation set, validation set, and combined populations.

The AUROC for these data was 0.83 (95% CI, 0.78–0.89), indicating that the model is an excellent predictor of mortality (Figure 2). The results of Hosmer-Lemshow chi-squared testing (*P = *0.17) were indicative of good calibration. The prediction rules derived using this scoring system are listed in Table 5 for different thresholds, with the associated sensitivities, specificities, positive and negative predictive values, and overall accuracy. Using a cut-off score of 3 points to discriminate between high-risk and low-risk patients, the scoring system was found to have a sensitivity of 58%, a specificity of 90%, a positive predictive value of 61%, a negative predictive value of 89%, and an overall accuracy of 83%. Patients with scores of ≥3 points had an OR for mortality of 5.12 (95% CI 5.75–27.85, *P*<0.001).

**Table 5:** Model and risk score performance: derivation set (n = 247) and validation set (n = 247).

	TP	FP	TN	FN	Se	Sp	PPV	NPV	Acc
**Derivation set**									
Score ≥ 1	52	165	30	0	100	15	24	100	33
Score ≥ 2	47	72	123	5	90	63	39	96	69
Score ≥ 3	30	19	176	22	58	90	61	89	83
Score ≥ 4	8	5	190	44	15	97	62	81	80
Score = 5	1	0	195	51	2	100	100	79	79
**Validation set**									
Score ≥ 1	30	204	13	0	100	6	13	100	17
Score ≥ 2	30	121	96	0	100	44	20	100	51
Score ≥ 3	25	13	204	5	83	94	66	98	93
Score ≥ 4	16	3	214	14	53	99	84	94	93
Score = 5	3	0	217	27	10	100	100	89	89

Abbreviations: TP, number of true positives; FP, number of false positives; FN, number of false negatives; TN, number of true negatives; Se, sensitivity; Sp, specificity; PPV, positive predictive value; NPV, negative predictive value; Acc, rate of accuracy of the risk score model.

The unequal-weight model, which assigned a different weight to each risk factor based on the multivariable logistic regression coefficient, had the same AUROC values and similar calibration for both the cohorts; because the equal-weight risk score model was easier to apply than the unequal model, we chose to report on the first model only.

#### Validation set

The prediction rules derived from the scoring system in the validation set are listed in Table 5 with the prognostic performance parameters for the main cut-offs. The ORs for mortality were even higher than those observed in the derivation cohort: 78.46 (95% CI 23.50–293.09, *P*<0.001) for scores >3. As shown Figure 2, when the prediction rule was applied in the validation cohort, the model once again exhibited excellent predictive power (AUROC 0.95; 95% CI, 0.89–1.00) and good calibration (Hosmer-Lemshow *P* = 0.75).

#### Application of the model in the combined cohort

When we combined the two cohorts (n = 494), the predictive effects of the model were similar to those observed in the derivation and validation sets. The ORs for mortality associated with a score of ≥3 was 24.18 (95% CI 12.94–45.33, *P*<0.001). The 3 cut-off displayed a sensitivity, specificity, PPV, NPV, and an overall accuracy of 67%, 92%, 63%, 93%, and 88%, respectively. In the combined cohort, the prediction rule had an AUROC of 0.86 (95% CI, 0.83–0.89) (Figure 2).

## Discussion

A score that can be used to predict the likelihood of mortality is useful in the evaluation of patients with severe infections because it offers criteria to use when choosing a clinical management strategy. In high-risk populations, such as those admitted to intensive care units (ICUs), several scores are already widely used to predict clinical outcomes [20]–[22]. However, published studies incorporating analyses that establish a risk score for mortality in patients with HMs are scarce and mostly based on adult or pediatric patients in the setting of ICU admissions or on patients who developed febrile neutropenia [23]–[26].

To the best of our knowledge, there have been no publications to date reporting the analysis of mortality-based scores in adult non-ICU patients affected by HMs with concurrent BBSI. We have developed and validated an easy-to-use risk stratification tool that is based on five variables that were found to be independently associated with mortality in a population of 494 patients.

Our study was conducted in nine hematological centers that regularly admit high numbers of patients with HMs. The possible confounding effects of different types of HMs and/or various stages of treatment were minimized by matching patients in the derivation and validation cohorts according to these parameters.

The multivariate model identified five factors associated with a higher mortality for patients with BBSI and HMs. These include acute renal failure, severe neutropenia (ANC <100 cells/mm^3^), nosocomial infection, age ≥65 years, and Charlson Comorbidity Index ≥4.

Our score is simple to calculate and is constructed from variables that are readily available at the time of admission and can be generated from the demographic characteristics, elements of the patient history, and routine clinical findings. This score provided good discrimination of mortality risk in both the derivation and validation sets, with AUROCs indicative of an excellent predictive power. Furthermore, the fact that the patients included in the validation cohort came from 8 different hospitals and were hospitalized during different time periods increases the likelihood that our findings can be generalized to a broad range of patients with HMs.

The overall mortality rate was lower (12.1%) in the validation cohort than in the derivation cohort (20.6%); this finding is important considering the better global performance of this score in the validation cohort. In addition, the prevalence of Gram-negative agents, which are usually associated with a worse outcome, is significantly higher in the validation cohort than in the derivation cohort; this result reinforces the predictive value of the score independent of the etiological agents responsible for the BBSI.

When a threshold of ≥3 was used, the specificity of prediction was over 90% in the derivation set and 94% in the validation set. Although sensitivity was relatively low at 58% in the derivation set, it reaches 83% in the validation set, and the high specificity of the prediction could improve the targeting of patients with a higher mortality risk.

Decisions related to the initiation of more intensive care treatments are challenging, especially when they concern patients with cancer. While the decision to admit patients with cancer to ICUs is difficult to make and should be based on the prognosis for each patient, the application of close patient monitoring and life-support measures should be implemented in all patients with BBSI and a high risk of mortality (i.e., ≥3 points with our score).

In the updated clinical practice guideline for the use of antimicrobial therapy in neutropenic patients with cancer developed by the Infectious Diseases Society of America (IDSA), two different risk classifications have been proposed for identifying high-risk patients: the first is based on expert opinion and identifies high-risk patients as those with anticipated prolonged (≥7 days duration) and profound neutropenia (ANC <100 cells/mm^3^ following cytotoxic chemotherapy) and/or significant medical co-morbidities; the second classification risk proposed is the Multinational Association for Supportive Care in Cancer (MASCC) scoring system, with a cut-off ≤21 to identify high-risk patients [27].

The MASCC scoring system, which had been validated only for patients with febrile neutropenia and cancer, has been subsequently applied in a cohort of bacteremic patients with cancer, confirming an approximate correlation between score and risk of complications and death; however, no stratifications have been made between patients with HMs and solid cancers (who could have different characteristics, for example, regarding the role of neutropenia in the development and outcome of BBSIs) [28]. In addition, as highlighted in the updated IDSA guidelines [27], a fundamental difficulty with the MASCC system is the lack of a clear standardized definition of one of its major criteria, i.e., the “burden of febrile neutropenia” and symptoms associated with that burden, which might complicate the uniform application of the MASCC tool.

Our scoring system has been developed specifically for hospitalized patients with HMs and concurrent BBSI, and it can be applied soon after BBSI onset. For this reason, it could be used for risk assessment in the setting of patients with HMs and BBSI, as well as in association with the classifications proposed in the IDSA guidelines for the management of patients with febrile neutropenia and cancer.

Finally, inappropriate antimicrobial therapy during the empirical phase of treatment has been well demonstrated as the main risk factor for mortality in non-hematological patients with BBSI [29], [30], and the same finding has also been reported in patients with HMs [12], [13]. The use of the present scoring system could provide useful information for prescribing a more broad spectrum empirical therapy according to individual (i.e., previous colonization and/or infections) and local bacterial epidemiology in patients with scores ≥3, until microbiological data become available.

It is important to note that the application of the scoring system in empirical treatment decision-making processes needs further validation to quantify its value as a risk assessment tool compared with the clinical judgment of hospitalists, which is likely to be variable from one setting to another.

In conclusion, we stress that BBSI in patients with HMs is a frequently observed clinical condition that requires prompt recognition and treatment with adequate antibacterial therapy, with consideration of the increasing number of multi-drug resistant etiological agents. Our results have demonstrated that patients with BBSI at higher levels of mortality risk can be reliably identified by the application of a simple clinical prediction rule based on five easy-to-define variables that are readily available at the time of BBSI onset. This type of risk stratification could be an important strategy for improving clinical decision-making in high-risk patients, such as patients with HMs.
